# Multidimensional evaluation of teaching strategies adopted in the COVID-19 pandemic

**DOI:** 10.1007/s10209-022-00954-z

**Published:** 2022-12-09

**Authors:** Rafael Molina-Carmona, Carlos Guillem

**Affiliations:** 1 Smart Learning Research Group, University of Alicante, 03080 Alicante, Spain; 2 Communication Office, University of Alicante, 03080 Alicante, Spain

**Keywords:** Teaching strategy, Sustainability, Usability, Accessibility, Evaluation model, Multidimensional evaluation

## Abstract

This paper proposes a multidimensional social open model to evaluate the teaching strategies adopted during the COVID-19 pandemic by assessing the decisions made by teachers by a group of teachers acting as evaluators. Based on the analysis of previous studies on teaching, this study aims to propose a formal model for the evaluation of teaching strategies in four dimensions: sustainability, usability, accessibility, and creativity. The use of information technologies to measure teaching strategies can bring decisive advantages. This work has been inspired by social rating systems of social networks to propose a measurement system in which a potential large number of evaluators with different levels assess the strategies. In addition, the proposal also includes the evaluation of the evaluators' own work, assigning confidence levels that are based on their experience but also on their evaluations. In this way, we have a social measurement system, in the sense that participation is open to a large number of evaluators. A large community of teacher evaluators will increase the objectivity of the measurement. The outcome of the system will be a characterization of the teaching strategies that will allow to decide in the future which ones should be adopted according to the needs of each one.

## Introduction

Since the beginning of 2020, with the arrival of the COVID-19 pandemic, we have lived through a time of change, a time in which new problems and difficulties appear to provide solutions that were frequently given in a certain way. In record time, society in general and the university community in particular have had to resort to ingenuity and creativity to be able to continue providing the same or almost the same response as before. In some cases, the new solutions provide a much greater potential than expected.

The adaptations in education due to the pandemic have been quite similar all over the world, although some teachers have tried to provide their own solutions individually and others have searched solutions in association with other colleagues. Most successful solutions, with a clear commitment to digitalization, have come in a social and cooperative way, since the collectives with similar problems in some way have sought to associate in order to become stronger and face the problems in a successful way. This emergency situation has led us to verify the strength of the group in creative processes and the potential for improvement when working together.

Despite the success and growth of a lot of digital tools that may settle down after the pandemic, many others may not. Once classroom attendance has been resumed and a certain degree of normality has returned, we may wonder what will be left of all that, which digitized processes will remain and which will be abandoned and, finally, if there will be a true digital transformation of education or whether the digitization process will be reversed after some time. The primary objective of this research is to shed some light on these questions by evaluating the main teaching strategies adopted during this period.

To assess learning strategies, we must address three aspects: (1) select the learning strategies we want to evaluate, choosing the most important and most commonly used in higher education, (2) choose the criteria that will facilitate this evaluation, so that it can be synthesized and interpreted correctly, (3) construct an evaluation instrument appropriate to the problem," since it will provide objectivity to the evaluation of the strategies. The selection of the teaching strategies to be analyzed was made by means of a literature review that allowed us to identify the main solutions adopted by teachers during the pandemic. As for the criteria for evaluation, we have established four: sustainability, usability, accessibility and creativity of the adopted strategy. Finally, we have adapted an evaluation model based on the social participation of experts that we had previously described to evaluate the creativity of products and their creators, adapted in this case to teaching strategies.

The article is structured as follows: in Sect. 2, we present a review of the literature on rapid adaptation of teaching to the COVID-19 crisis and on social assessment, brief but focused on the key aspects necessary to understand our model. In Sect. 3, we explain what the developed model consists of. The evaluation of teaching strategies with the adaptation of the model is presented in Sect. 4. The application of the evaluation model to the case of teaching strategies and its results are described in Sect. 5. The conclusions are presented in Sect. 6.

## Background

There are a lot of research works on the perceived success and failure of teaching strategies, but we want to go deeper into each of them and evaluate them, in the context in which they were quickly adapted to use, to determine whether they were effective by taking advantage of a comprehensive measurement model in which we break down the attributes of each dimension that we want to analyze. This study is limited to the university environment, which has special characteristics that differentiate it from primary and secondary education.

In this section, we present the main background of our research: first, a review of the adoption of teaching strategies during the COVID-19 pandemic, to help us identify what these strategies have been and their main advantages and disadvantages. This is followed by a brief presentation of social evaluation systems, which have served as inspiration for the evaluation model we have used.

### Rapid adaptation of teaching to the COVID-19 crisis

The COVID-19 pandemic took organizations by surprise. All institutions and, particularly, universities had to close their doors and opt for virtuality in their processes, teaching their classes thanks to digital technologies [1]. Universities solved the unexpected with the use of online platforms to develop their organizational and educational processes [2]. In record time, a regular activity was transferred to a new dimension for which both teachers and students could be prepared or not. Teachers who had never taught e-learning before were forced to use this type of system to develop their classes, which made it necessary to learn not only the working model, but also the ICT tools [3].

All this technology that was used already existed and was more or less implemented and part of a progress in teaching, but it had to be used by the whole community and in all situations, which was a real revolution. Not just technologies but notably teaching strategies underwent this revolution, largely driven by the availability of technology, which has never been as mature as it is today. Current technological teaching platforms and virtual classrooms allow the use of countless tools for interaction between the agents of the teaching–learning process (teachers and students), including presentations, videos, chats, social networks, evaluation activities, delivery of exercises, practices, reports and presentation of learning results. However, before the pandemic, its implementation was generally limited to a basic use, since the pedagogical model was still that of the teacher as a transmitter of content in a face-to-face session [4].

Teaching methodologies and strategies are not necessarily linked to technological development, but indeed it is a fact that this development has led in many cases to the spread of these methodologies and strategies, and even to the emergence of new ones. The pandemic has meant that many methodologies and strategies have started to be used in a very recurrent way during this period: flipped classroom [5], project-based learning [6], cooperative learning [7], gamification [8], problem-based learning [9], among others. Moreover, another debate that has been opened, relates to synchronous or asynchronous teaching, with the former standing out for its agility and interaction, and the latter for its autonomy and availability. Advantages and disadvantages of synchronous and asynchronous online teaching are discussed in the work of Contreras et al. [10].

The change in teaching methodologies and the lack of physical presence have inevitably led to a change in the assessment methods. The evaluation systems had to adapt to this situation, implementing different options of online test-type questionnaires and videoconference tests [11]. This circumstance developed doubts among teachers about the reliability of distance assessment tests [12]. Among the proposals and suggestions for evaluating university students during the COVID-19 pandemic, the use of oral tests via videoconferencing platforms is one of those recommended for subjects with a small number of students [11].

All these forced and sudden changes have not been at zero cost. The COVID-19 pandemic forced the university community to redouble its efforts; dealing with virtual teaching demanded a great deal of dedication from professors and students. Most teachers had never taught e-learning [13] and many did not even feel prepared to teach this type of education due to their limitation in the use of ICT, an essential tool whose proper implementation depends on the existence of digitally competent teachers [14].

The teachers have had to improvise in the adoption of teaching measures, which has hardly allowed them to reflect and transform their didactic proposals to respond to the demands of a world affected by a health crisis, while achieving the curricular objectives proposed at the beginning of the course [15]. In short, the quality of teaching has been compromised [16].

There was also a great deal of difficulty in adaptation among the students, even among those who were supposed to be better prepared for this sudden change: "they felt stressed by the uncertainty of having to adapt to a model they were not used to and which demanded greater commitment and discipline" [17].

Everything points to the fact that although the idea behind the response was good, the adaptation process was necessary and could not be carried out, so that general satisfaction was not high and a return to pre-pandemic teaching was called for when possible. For Compañ-Rosique and Satorre-Cuerda [18], the results obtained in traditional teaching are not maintained according to the teachers' perception due to the lack of interaction in the classroom. It is observed that teachers perceive more advantages while students observe a greater number of disadvantages with the virtualization of their classes. However, despite recognizing that they learn more with the face-to-face system, students have opted mostly for the virtual teaching options. Finally, the students recognize that the system that best evaluates their knowledge is the face-to-face tests [1].

Other studies related to the evaluation of teaching quality during COVID-19 focused on other aspects; for example, the work of Hurtado et al. [19] focused on student interaction, concentration during online classes, online test review, system usability and diversity of evaluation activities.

Another interesting article is that of Sanchez Gonzalez et al. [20] in which teaching strategies are evaluated based on four items: reliability, validity, trustworthiness and variance.

### Social evaluation

Social rating systems are an inspiration for social evaluation of products. A social rating system can be defined as a mechanism or tool that allows the evaluation of a product, in the broadest sense, based on the opinions of a large group of people that we will call evaluators.

Social networks have introduced mechanisms such as “likes” to evaluate products, very simple but very effective type of surveys. One example of system that offers the option of giving a positive vote to the product is that of the social networks Facebook [21] or Instagram [22]. In this case, there is no possibility of quantity, quality or negative votes. There is a more enriched variant of the previous rating system. It allows a positive or negative rating of the product by offering “Like”/“Dislike” options. An example service that uses this type of ratings is YouTube [23].

Another possibility is that of systems that rate the product on a scale of values. For instance, [24] uses a rating system based on “Stars,” positive values that are accumulated and averaged. Other systems use scales based on the widespread Likert scale [25], so that the scores of the users can be negative, neutral or positive. The rating system used by TripAdvisor [26] is also an average, but other factors such as time or text comments are also considered. This increases the complexity of the rating system but the information is richer. Finally, another case is that of a classic evaluation from 0 to 10 where the value is updated averaging different user ratings. These ratings are common in video or music streaming portals [27].

Most platforms do not consider the trajectory or behavior of evaluators to weigh their votes. Due to this, the final score is subjected to the good faith and the ethics of the users. Therefore, there is the possibility of sabotaging the ratings. To avoid this risk of sabotage, new strategies have appeared in the context of social networks and e-commerce platforms. They are based on two concepts: reputation and trust. The concept of reputation is defined by Kietzmann [28] as “the extent to which users can identify the standing of others, including themselves, in a social media setting”. Sztompka [29] states that “trust is a bet about the future contingent actions of others”. Golbeck [30] considers that there are two main components of trust: belief and commitment. First, a person believes that the trusted person will act in a certain way. However, the belief is not enough to say there is trust. Trust occurs when that belief is used as the foundation for making a commitment to a particular action.

The issue of trust and reputation on the web has been studied since the origins of the web, but today, with the flowering of the social networks and the e-commerce platforms it is a hot topic. Most platforms have introduced methods for rating the reputation of sites or users [31].

Inspired by social rating systems and the concepts of trust and reputation, in 2018 we presented a social evaluation model for creative products [32, 33]. The model is based on the idea that a product is the result of the activity of a creative individual and, therefore, the tangible element resulting from his/her creativity. In this way, the product is the element of the model that is evaluated in such a way that the creative individual is characterized by the assessment of his or her products. In order to carry out these evaluations the participation of the evaluators, the other fundamental participant in the proposed measurement model is needed. At the same time, the evaluators are characterized by the quality of their evaluations, demonstrated throughout their participation in the system. A deeper explanation of the model is presented in the next section.

## A social and open model of evaluation

The social and open model for measuring creativity [33] is based on the idea that a product is the result of the activity of a creative individual and, therefore, the tangible element resulting from his creativity. In this way, the product (it can be an object, an idea, a service, a process, or any result of a creative process) is evaluated with the participation of experts that make up the social aspect of the model. The experts evaluate a set of variables or facets, grouped into dimensions, and this evaluation of product facets provides a measure of product creativity, as well as a characterization of the experts themselves. The possibility of defining the dimensions and facets specifically for the product we want to evaluate is the open part of the model.

Although the model was created to measure product creativity, its open nature facilitates its use to measure and evaluate other attributes that may be of interest to us. In this case, we will describe the model for use as an evaluator of different characteristics associated with teaching strategies. We will first explain the elements that are part of the model and then the dynamics of the system which produces the final evaluation.

### Elements of the evaluation model

To explain the model, the first step is to define the main elements that are part of it. Later sections explain the relationships between these elements and the dynamics of the model. Accordingly, the main elements are:

- Product: The product is the object of evaluation. It can be any object, idea, service, process or, as in our research, teaching strategy. It is characterized by a set of facets.
- Facet: The facets are the attributes or features of the product that can be measured and which indicate the extent to which the product has the studied feature. The facets are grouped into dimensions.
- Dimension: The dimensions are facet groups that define the general characteristics of the product.
- Creator: The creators are the individuals who perform the process and create the product as a result of that process. The attributes of the product will also characterize the individuals who created it. The evaluation of the attributes of the individuals is calculated from the evaluation of the products they have created.
- Evaluator: Evaluator is the expert who evaluates the products through an evaluation questionnaire. In turn, the proposed model allows the evaluation and characterization of the work of the evaluators. Most measurement models assume that evaluators are subject matter experts. However, in some studies, such as Besemer's [32], any user is worthy of being evaluated, thus facilitating the measurement of evidence as it is often difficult to find experts available and willing to perform evaluations. That is why a so-called confidence level is assigned to each evaluator to characterize them.
- Confidence level: In our model, evaluators are ranked according to their level of experience and their degree of success with the resulting consensus in each evaluation in which they participate. The model classifies evaluators into 5 levels of confidence according to their training, experience, academic rank and the quality of their evaluations. Evaluations performed by a higher-level evaluator are more influential than those of a lower level, on a defined scale. It is the model itself that is responsible for presenting the product to be evaluated to different evaluators of different confidence levels.

### Dynamics of the model

**Fig. 1:**
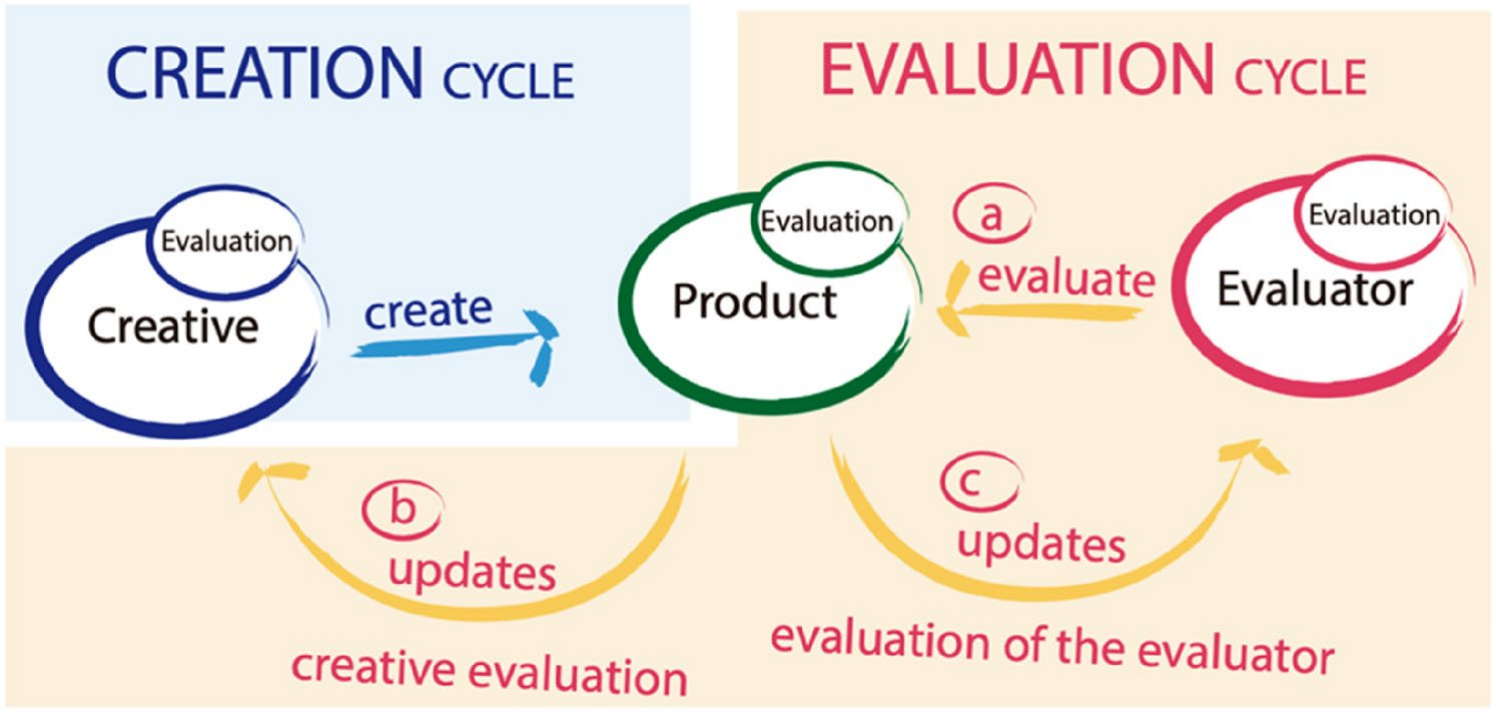
Dynamics of the model

To complete the general explanation of the model, it is important to explain the dynamics of the system. The model works iteratively, and it is formed by two cycles: the one of creation and the other one of evaluation (Fig. 1):

- Creation cycle: the creator generates a product and makes it available to the evaluation system. The system remains on the look-out for new products that are created.
- Evaluation cycle: when there is a product to evaluate, the evaluation cycle begins, which in turn consists of the following steps:
  - Evaluate: the evaluator evaluates the product according to an evaluation procedure that will be explained later. The result of this procedure is incorporated as an evaluation of the product;
  - Update the creator evaluation: the result of the evaluation of the product contributes to the evaluation of its creator, in the form that is explained below;
  - Update the evaluator evaluation: the result of the evaluation of the product contributes to the evaluation of the evaluator.

In short, after performing the different cycles with the work of the different creators and evaluators, an evaluation for each of these three elements is obtained.

### Evaluation procedure in detail

This section presents how the products are evaluated and how the levels of confidence of the evaluators are updated, that is, how the evaluators are assessed themselves.

#### Evaluation of facets

Each facet is classified in one of the dimensions considered in the model, having a total of n facets, so that we will formulate n different questions so that each facet is evaluated in a Likert scale from 1 to 5. Thus, the score of a facet is the mean of the score given by the evaluators, weighted by the confidence level of the evaluators (the higher the level, the higher the weight). More formally, given a product *p ∈ P* (*P* is the set of all the products), we define the set F of *n* facets to be evaluated, and a set E of *m* evaluators. Each evaluator *e*_*j*_ ∈ *E* is assigned a confidence level *l*_*j*_ ∈ {1, 2, 3, 4, 5}. In addition, given a facet *f*_*i*_ ∈ F, we define *E*_*i*_ ⊂ E as the subset of evaluators that have evaluated the facet f_i_. For each evaluator *e*_*j*_ ∈ *E*_*i*_, we define *v*_ij_ ∈ {1, 2, 3, 4, 5} as the evaluation given to facet *f*_*i*_ by the evaluator *e*_*j*_. Finally, we can define the evaluation *v*_*i*_ of the facet *f*_*i*_ as:

1 $${v}_{i}=\frac{{\sum }_{\forall {e}_{j}\in {E}_{j}}{v}_{\mathrm{ij}}\bullet {l}_{j}}{{\sum }_{\forall {e}_{j}\in {E}_{j}}{l}_{j}}$$

#### Update of evaluator confidence levels

The confidence level of an evaluator represents how good this evaluator is at evaluating the quality of the product, that is, the evaluation of the evaluator. Since we consider that the evaluation v_i_ of the facet f_i_ of a product p obtained from Eq. 1 is a good approximation to the actual quality level of each facet of the product, we can consider that an evaluator is better as his or her evaluations are closer to this canonical evaluation v_i_ (we consider this evaluation to be canonical due to the fact that in the model it is accepted to be the right and agreed evaluation of the facet). We formally define the difference *d*_*j*_ between the evaluation *v*_*ij*_ of a given evaluator e_j_ for a product and the canonical evaluation *v*_*i*_ of the same product as:

2 $${d}_{\mathrm{jk}}=\frac{\sum_{\forall {f}_{i}\in F}\left|{v}_{\mathrm{ij}}-{v}_{\mathrm{ik}}\right|}{n}$$

where *F* is the set of facets and *n* the number of facets.

Now, we must define the confidence degree *g*_*j*_ of an evaluator *e*_*j*_ as a real value in the interval [1, 5] that estimates the quality of his or her evaluations. The confidence degree is a continuous version of the confidence level of the evaluator *l*_*j*_, so that *l*_*j*_ = round(*g*_*j*_). The confidence degree of an evaluator is initialized at instant 0 with a first approximation of the confidence level given by the managers of the system. Typically, this value is obtained from the expertise of the evaluators, so that experts in quality should be assigned a level of 5, and novices in this field a level of 1. The confidence degree is now on updated for every new evaluation, depending on how good the evaluator at evaluating is. In other words, if the difference *d*_*j*_ between his or her evaluation and the canonical one is close to 0, the evaluator is accurate and his or her confidence degree should be incremented. On the contrary, if the *d*_*j*_ has a high value, the confidence degree should be decremented. To do so, we propose classifying this difference into three possible ranges, so that the confidence degree is updated in a quantity depending on the value of d_j_:

3 $${u}_{j}=\left\{\begin{array}{c}0.1\mathrm{ \;if\; }{d}_{j}\le 0.5\\ 0\mathrm{\; if \;}0.5<{d}_{j}\le 1\\ -0.1\mathrm{ \;if \;}{d}_{j}>1\end{array}\right.$$

Now, the confidence degree of the evaluator *g*_*j*_ can be recursively defined using the following equation:

4 $$\left\{\begin{array}{c}{g}_{j}^{(1)}={l}_{j}^{(0)}\\ {g}_{j}^{(k)}={g}_{j}^{(k-1)}+{u}_{j}\end{array}\right.$$

The confidence degree assigned to evaluator *e*_*j*_ at instant 1 is the initial assignment (denoted *l*_*j*_^*(0*)^) given by the managers of the system.

Finally, the confidence level at instant *k* is obtained just rounding the confidence degree:

5 $${l}_{j}^{(k)}=\mathrm{round}\left({g}_{j}^{(k)}\right)$$

### Evaluation of creative products

As already mentioned, this model was created with the intention of measuring the creativity of the products. In this section we present a brief example of this application, which serves to compare it with its use to evaluate teaching strategies, which is the objective of this article, and which will be presented in the following section.

Our social and technological model of creativity evaluation was based on 3 dimensions, broken down in turn into 66 facets. The validation of the model and a real use case on the evaluation of three creative products are presented in Guillem-Aldave and Molina-Carmona [33, 34]. This type of classification by dimensions and facets of creativity measurement finds its origin in other studies of creativity. The most outstanding reference in this work is found in the figure of Susan Besemer and Treffinger [35] that was the basis of our proposal. They present their creative product analysis matrix, which proposes three dimensions (novelty, resolution and style) and nine subscales, equivalent to our facets (surprising, original, logical, useful, valuable, understandable, organic, well-crafted and elegant).

However, there is extensive research on the literature on measuring the creativity of a product, and options of dimensions and facets that characterize these products. For instance, Sternberg and Lubart [36] consider that a product can be defined as creative when it is original and appropriate. Along the same lines, Amabile [37] states that the product must be appropriate, useful and correct. Romo Santos [38] establishes three interesting factors that help determine the value and quality criteria of a product: transformation (making new combinations or different formulations of what already exists), condensation (relating and agglutinating a large amount of information that has never been linked before) and the area of applicability (the product must generate additional creative activity).

Although the initial model was designed and based on the study of the measurement of creativity in products, we consider that it can be extrapolated to other disciplines and characteristics and this study is a good opportunity to demonstrate it.

## Model adaptation to evaluate teaching strategies

The aim of this paper is to evaluate the teaching strategies adopted by teachers during the COVID-19 pandemic according to their sustainability, usability, accessibility and creativity. The social and open model of evaluation that we have defined is well suited to this task for two main reasons. On the one hand, it is an open model that allows to evaluate any product according to a set of attributes, also definable upon the needs of each particular case. Therefore, it is perfectly suitable for the evaluation of teaching strategies based on the defined attributes. On the other hand, it is a social model, that is, it allows the participation of as many evaluators as desired, belonging to different levels of expertise, who evaluate teaching strategies collaboratively. This last issue is relevant, since the development of the strategies themselves has also been an effort of many teachers working collaboratively, and what better than to evaluate their results jointly.

### Teaching strategies

The teaching strategies applied during the pandemic have been numerous. Different authors have described different strategies, some very different and others similar. To determine which strategies were to be included in this study, we defined the following criteria:

- The study involved the participation of as many expert evaluators as possible. In order to gain access to these experts and to achieve a thoughtful but rapid evaluation of the strategies, it was important that the set of these strategies be limited, bearing in mind that each strategy must be evaluated for each attribute to be considered. We proposed to handle no more than 10 strategies.
- In the literature, the definition of strategies is not homogeneous, and there are many that differ only in small nuances. A widely used and generic definition of strategies is sought to accommodate all those strategies that are very similar.
- The strategies chosen were to be the most widely used. To this end, the existing literature was extensively reviewed, a summary of which is provided in the background section.
- Although we speak of teaching strategies, these include methodologies for both the delivery of classes and the learning process, as well as for the evaluation of this learning. For this reason, the chosen strategies had to consider methodologies for the entire teaching/learning process, including evaluation.

As a result of applying these criteria, the following strategies have been chosen for this study:

- Synchronous master class: Master class taught in real time through a videoconferencing application;
- Asynchronous master class: Master class that is recorded in video format and made available for students to view at their convenience;
- Training pills: Recorded contents in video format that are characterized by dealing with specific topics and have a very short duration. They are made available for students to view at their convenience;
- Flipped classroom: Synchronous class, in this case by videoconference, which is taught once the students have previously worked on the contents of the subject on their own;
- Project/problem-based learning: Type of learning that is developed from a project or problem that the student must work on his own, in a group or individually, supervised by the teacher;
- Synchronous exam-based assessment: Traditional assessment test, usually an exam-type test, in which students are monitored remotely, with or without video surveillance;
- Asynchronous multimodal assessment: Assessment through several tests and assignments of different types, without remote monitoring;
- Project/problem-based assessment: A type of evaluation based on the results of a project or assignment, which does not include an exam-type test;
- Oral assessment: Oral defense assessment, individual or group, conducted by videoconference.

### Dimensions and facets

Once the teaching strategies to be evaluated have been defined, it is necessary to determine the dimensions and facets that make up these dimensions. The dimensions are the four we have already defined: sustainability, usability, accessibility and creativity. These four dimensions consider the main factors that may influence the future use of these strategies once we return to face-to-face teaching. Based on these four dimensions, we have proposed two facets per dimension, that is, eight facets in total that allow an evaluation of the strategies based on specific attributes. A justification of the dimensions and facets is presented below, along with a summary in Table 1.

**Table 1:** Dimensions and facets of our model

Sustainability	Usability	Accessibility	Creativity
More work	Teacher training	Accessible for disabled students	Innovative
More resources	Student training	Accessible for general students	Useful

For a teaching strategy to remain in the future, we have considered that it must be *sustainable* over time, that is, the work required to maintain it must be limited and not consume more resources than the strategy it replaces. Furthermore, it must be easily *usable*, not requiring specific training on the part of the teacher or the student to be able to use it. Moreover, it must also be *accessible*, in other words, it must be easy to access by all the diversity of users, particularly by users with disabilities. Finally, we believe that it must also denote a high level of *creativity*, so that it provides innovative and surprising solutions for the user.

## Evaluation of teaching strategies

The study was carried out with the participation of 34 expert professors, belonging to 9 universities, distributed among the following fields of knowledge:

- Arts and Humanities (1);
- Sciences (3);
- Health Sciences (3);
- Engineering and Architecture (18);
- Social and Legal Sciences (9).

These experts should be classified at the beginning in confidence levels, between 1 and 5, according to their training, experience and academic rank, because in the system the evaluations made by a higher-level evaluator are more influential than those of a lower level. This classification has been made by the researchers, with the distribution of experts by levels shown in Table 2.

**Table 2:** Number of experts for each confidence level

Level	Number of experts
1	4
2	7
3	9
4	6
5	8

Then, the dynamics of the system update these confidence levels according to the participation of each evaluator.

The experts have evaluated the 9 teaching strategies following the 8 facets according to the social and open model of evaluation. To do so, they were asked to rate the following 8 statements for each teaching strategy, on the Likert scale of 5 values shown in Table 3.

**Table 3:** Values of the Likert scale

Response	Value
Strongly disagree	1
Rather disagree	2
Neither agree nor disagree	3
Rather agree	4
Strongly agree	5

The 8 statements, proposed for each strategy, are as follows:

- This strategy requires more work on the part of the teacher;
- This strategy requires more resources to implement it;
- This strategy requires training on the part of the teacher;
- This strategy requires training on the part of the student;
- This strategy makes classes more accessible to students with disabilities;
- This strategy makes classes more accessible to students in general;
- I consider this strategy to be innovative;
- I consider this strategy to be very useful for efficient learning.

When interpreting the results, we should bear in mind that the first two dimensions (sustainability and usability) are asked with a negative co-notation, that is, a higher value in the four facets corresponding to these dimensions implies a lower value in these facets. However, in the case of accessibility and creativity, the answers are in a positive way. In order to make comparisons, the values corresponding to the first two dimensions have been inverted, so that the four dimensions are always positive.

**Fig. 2:**
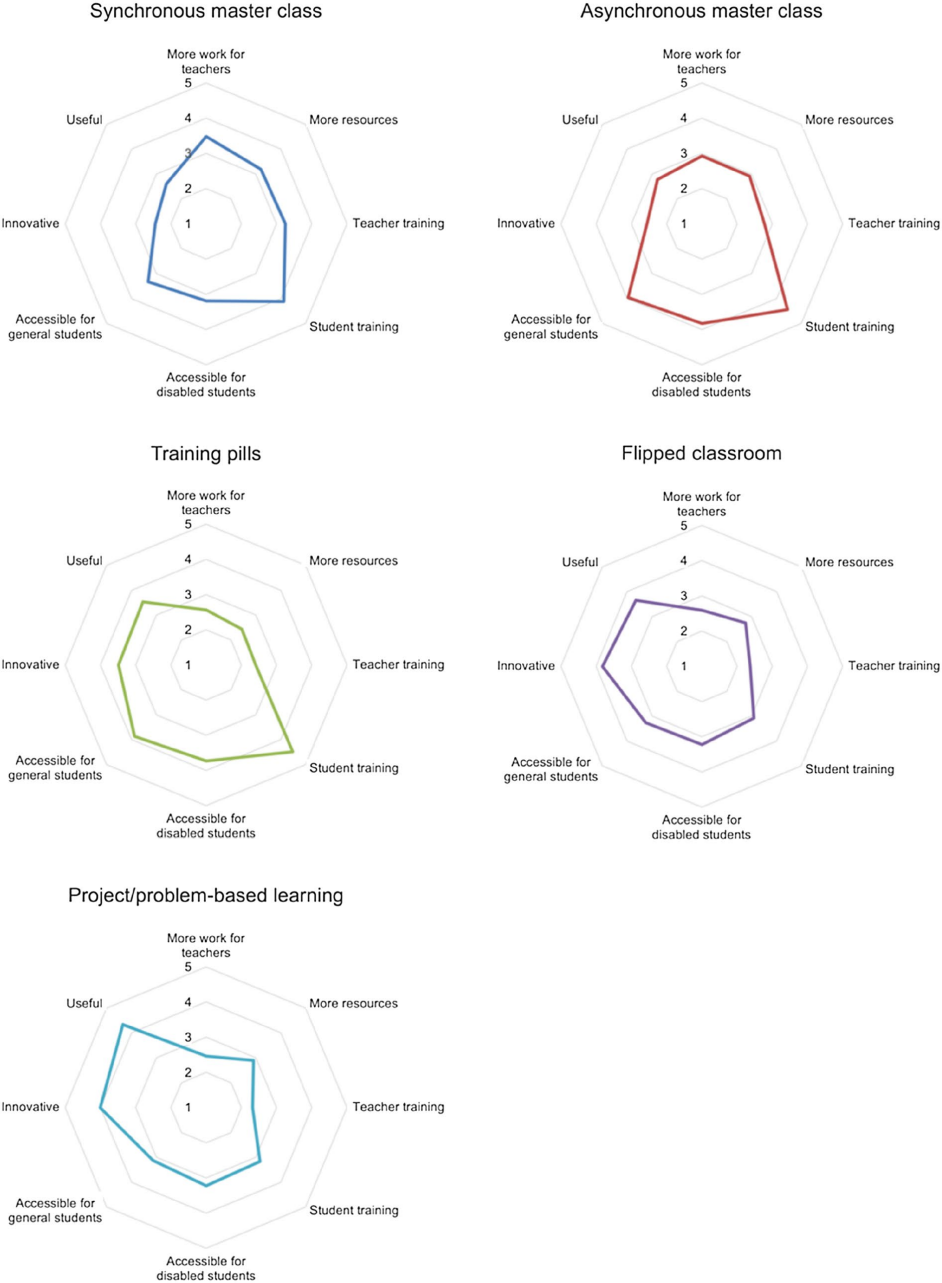
Facet evaluation for lecture delivery and learning strategies

Figure 2 shows the results of the evaluation of the 8 facets for the 5 lecture delivery and learning strategies, in the form of radial graphs. The synchronous master class strategy, which consists of transferring the lesson to a videoconferencing session, stands out for its sustainability and usability, in which it achieves the highest values among all class teaching strategies. However, it is among the least accessible and the least creative. These values should not be surprising since it is the one with the least variation over its face-to-face version.

As for the asynchronous master class strategy, simply recording the class and making it available to the students alters several of the facets under study. In particular, sustainability goes down because more work is required from the teacher, and accessibility goes up, since the possibility of providing the video with the recorded lecture offers very clear advantages for the student.

By taking it a step further and instead of providing videos with the complete lectures, we make short and specific videos available to the students, what are usually called training pills, the change in the evaluation of the strategy is much more significant. In this case, sustainability drops radically, due to both the need for more work on the part of the teacher and the need for more resources. At the same time, perceived creativity rises significantly, so that this methodology is seen as both innovative and useful. The usability and accessibility values do not change substantially with respect to the previous strategy.

The other two strategies, flipped classroom and project or problem-based learning (PBL), are active methodologies that put the student at the center of learning. Consistent with what the authors Llorens, Molina, Gallego and Villagra say [39] these strategies turn out to be more costly but more efficient in contexts such as the pandemic. Thus, in both cases the sustainability and usability dimensions are evaluated with a low score, due to the greater work required by the teacher and the need for training by both teachers and students. On the other hand, the creativity dimension is much higher than in the other cases, especially regarding the innovation and usefulness provided by PBL. In the case of accessibility, we believe that the greater diversity of tasks and their lack of predictability penalizes this dimension.

**Fig. 3:**
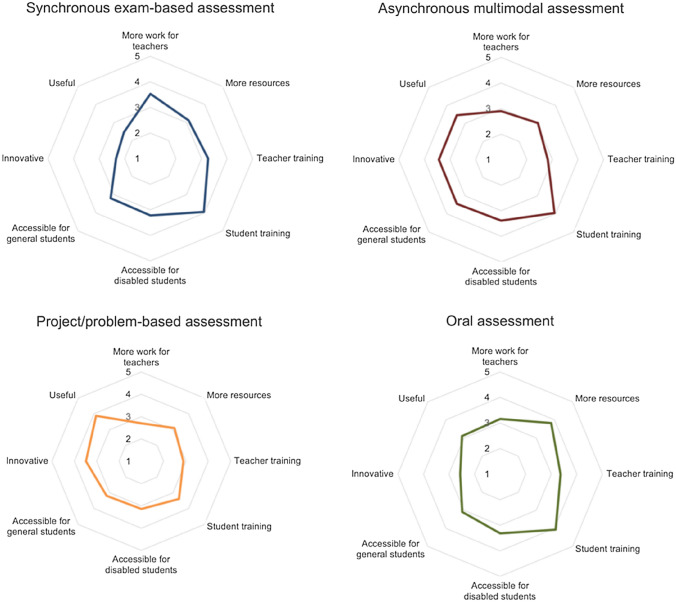
Facet evaluation for assessment strategies

As for the assessment strategies (Fig. 3), it can be observed that the synchronous exam-based assessment stands out for its sustainability and usability: as was the case with the more classic teaching strategies, its conversion to online is very simple and, therefore, does not compromise sustainability (it does not use many more resources and is not more laborious than its face-to-face version) or usability (neither teachers nor students need special training to use it). Accessibility, on the other hand, remains at a medium level, while creativity drops sharply. It should be noted that this strategy is the least innovative and, above all, by far the least useful of all the assessment strategies analyzed.

The asynchronous multimodal assessment strategy is one of the most balanced, standing out for its high accessibility (its multimodal nature favors the use of very varied assessment exercises that can be adapted to make them accessible) and good levels of creativity, being very innovative and, above all, very useful. In general, it does not compromise usability too much, although it does require teacher training.

As for sustainability, it remains at average values, with a significant but not overwhelming increase in the teacher's workload.

As might be expected, the project or problem-based evaluation strategy is the one with the most extreme rating in each dimension. It stands out, fundamentally, for its creative nature, being highly innovative and, especially, the experts consider this to be the most useful strategy of all. Accessibility and usability are, in general, below the values given to these dimensions for the other strategies, but they are not dramatically low. In this sense, it penalizes the fact that training is necessary, both for the teacher to successfully develop a methodology of this type and for the student to follow it adequately. Anyway, the lowest evaluation is for the sustainability dimension. It is a very demanding strategy, especially in terms of the level of work to be performed by the teacher.

Finally, the online oral assessment is perhaps that which changes the least with respect to the face-to-face oral assessment. Therefore, it stands out in terms of sustainability and usability, as it does not require any extra preparation or specialized training. The level of accessibility is medium, and the level of creativity is very low. In short, it is a conservative option that requires little additional work but is hardly innovative and has limited usefulness.

**Fig. 4:**
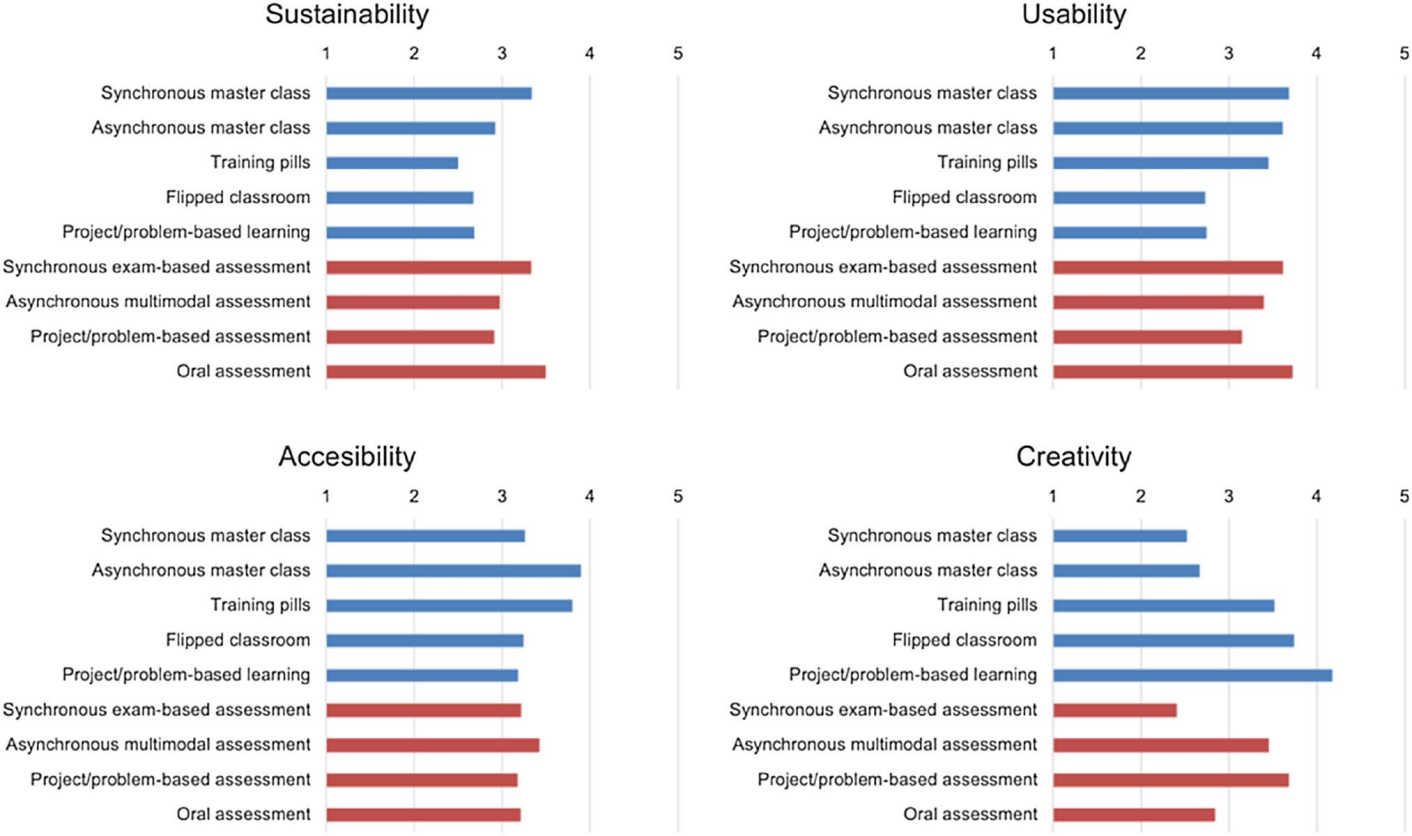
Comparison of the evaluation of the dimensions for each teaching strategy

The comparison by dimensions is also very interesting (Fig. 4). With respect to sustainability, strategy synchronous master class stands out as the most sustainable, since it is a transposition of the most classic strategy to an online environment. On the contrary, the least sustainable strategies are the training pills and the most active methodologies inverted classroom and PBL. If we look at assessment strategies, the situation is equivalent: the least costly (most sustainable) is to bring online the most classic forms of assessment, i.e., exams and oral tests. Multimodal assessment and project or problem-based assessment are, on the other hand, the least sustainable.

The creativity dimension, which measures the innovation and usefulness of the strategy, follows an almost inverse pattern to that of sustainability. The most sustainable strategies (more classical) are the least useful and innovative, while the most costly to implement (the most active methodologies) are the most innovative and, most importantly, the most useful for student learning. As a teaching and learning strategy, project or problem-based learning is particularly useful, and as an evaluation strategy, the use of projects and problems, as well as multimodal evaluation, also stand out.

The other two dimensions, usability and accessibility, have a more homogeneous behavior, with fewer differences between the different strategies. However, project- or problem-based learning and assessment and the flipped classroom are perceived as less usable than the more classical strategies, since they require previous training on the part of teachers and students. On the other hand, accessibility values are slightly better for classes that involve a video recording: asynchronous lectures and training pills. This may be due to the editing, transcription in subtitles and visualization possibilities offered by video applications.

## Conclusions

In this study, we have been able to verify that the teaching response during the COVID-19 pandemic was positive, with the use of strategies that were able to compensate quite acceptably for the difficulties encountered during this period. From the analysis, it is clear that several of the teaching strategies adopted were very well perceived by the teachers and it has been possible to obtain very detailed information on the strengths and weaknesses of each of them. This analysis helps us to select which digital strategies in teaching we can use as a basis for the future or in new emergency situations.

The evaluation of the strategies has been carried out by applying a social and open evaluation model, conveniently adapted to this problem. The application of this digital tool breaks down the information in a precise way, describing the attributes of the teaching strategies in terms of facets grouped into dimensions, which facilitates the evaluation by experts and provides as many points of view as facets are defined. The social nature of the tool allows the participation of experts of any level of expertise, and its open nature facilitates the introduction of new dimensions or facets. The tool also helps to assess the quality of the evaluations and to know how the evaluators behave, in which strategies there is more consensus and in which less, or which have been the most interesting of the strategies used.

In general terms, the dimensions of sustainability (work and resources required to implement the strategy) and usability to a lesser extent (prior training required) are inversely proportional to the dimension of creativity (innovation and usefulness of the strategy), that is to say, the strategies that are easiest to implement are usually the least innovative and useful. This can be easily verified if we compare traditional strategies directly adapted to an online environment (lectures and exam-type tests, whether synchronous or asynchronous) and active learning strategies (flipped classroom, PBL and multimodal assessment). The former require little adoption and training effort but have limited utility. The latter are much more costly in terms of effort, resources and training, but are much more useful and innovative.

The other dimension, accessibility, has a different behavior. In general, the use of video recordings and the availability of materials for offline review facilitate access to all users, especially those with disabilities.

The sample of our study was not very large, but it already reveals a lot of interesting information that could be expanded and analyzed in a subsequent study with a larger sample of participants. In addition, the body of expert evaluators can be easily enriched because the model allows for expansion without discarding the results obtained so far. The continuous use of the tool includes the quality assessment of the evaluation process itself and of the evaluators involved, which should lead to a rating of the evaluators' confidence levels based on facts and not on subjective appraisals.

## Data Availability

Data sharing is not applicable to this article as no datasets were generated or analyzed during the current study.
